# Effect of Modification and Hydrothermal Ageing on Properties of 3D-Printed Wood Flour–Poly(butylene succinate)–Poly(lactic acid) Biocomposites

**DOI:** 10.3390/polym15183697

**Published:** 2023-09-08

**Authors:** Wangwang Yu, Liwei Sun, Meihui Li, Youxue Peng, Chaohui Wei, Wen Lei, Rui Qiu, Ying Ge

**Affiliations:** 1 School of Mechanical Engineering, Nanjing Vocational University of Industry Technology, Nanjing 210023, China; 2 College of Science, Nanjing Forestry University, Nanjing 210037, China

**Keywords:** wood flour, poly(butylene succinate), poly(lactic acid), biocomposite, fused deposition molding 3D printing, hydrothermal aging

## Abstract

Wood flour–poly(butylene succinate)–poly(lactic acid) biocomposite samples were prepared by fused-deposition-molding 3D-printing technology, and modifications with glycerol and a silane coupling agent (KH550) were carried out. The samples were then hydrothermally aged. Modification with glycerol and KH550 enhanced the hydrophilicity of the samples and increased their tensile strength. Hydrothermal aging clearly whitened the surfaces of all the samples and made them more hydrophobic. Meanwhile, their tensile properties and thermal stability became poor; a higher hydrothermal aging temperature affected the mechanical properties more negatively. The modified samples turned out to be more resistant to the hydrothermal aging, and modification with KH550 could improve the anti-hydrothermal aging properties of the samples better than that with glycerol, where the tensile properties and the cross-sectional morphologies of the fractured specimens were concerned. Generally, the effects of hydrothermal aging temperature on the physico-mechanical properties of the printed specimens were greater than those by hydrothermal aging time.

## 1. Introduction

Three-dimensional (3D) printing, also known as additive manufacturing, is a rapidly evolving digital technology, enabling the automatic fabrication of frame structures with exact dimensions in a relatively short time for many applications such as civil, mining, automotive, aerospace, construction, foundry, and medicine [1,2,3]. Among 3D-printing technologies, fused deposition molding (FDM) 3D printing, also known as fused filament fabrication (FFF) technology, is the most popular and the least expensive, producing parts with no need for specific tooling.

Thermoplastic polymers, such as polylactic acid (PLA), acrylonitrile butadiene styrene (ABS), polypropylene (PP), polyethylene (PE), and polyphenylene sulfone are the main materials for FDM 3D printing [4,5,6]. In addition to the traditional polymer materials, more and more thermoplastic-polymer-based composites are being implemented in FDM applications [7,8]. Generally, the FDM-printed samples from the composites have some improved properties in comparison to those from the corresponding polymers. For example, polyamide (PA) is one important engineering polymer material; its overall performance is fine. However, its FDM-3D-printed products are easy to warp, distort, and deform. When some nano particles, such as graphene, silica, and montmorillonite are incorporated into PA, the shortcomings of pure PA during FDM 3D printing can be overcome and some nanocomposites with excellent properties and varied functions can be obtained [9]. Poly(lactic acid)(PLA)/talc composite was produced and used in FDM 3D printing by Yu et al. [10]. It was found that talc could act as nucleating agent in the matrix, and the introduction of talc thus increased the degree of crystallinity of PLA. Furthermore, the melt crystallization process released heat, promoting coalescence between adjacent layers, and made the composite parts have greater stiffness and better mechanical integrity than pure FDM PLA. Fu et al. [11] fabricated nylon and Al/nylon hybrid tubes using FDM 3D printing, then quasi-static axial crushing tests were carried out on the samples. The results showed that pure FDM nylon tubes easily underwent interlayer fracturing during folding, and thus were not desirable energy absorbing components, however, the printed Al/nylon66 hybrid tubes could avoid the adverse effects of this fracture and were suitable for energy absorption.

Due to their prominent advantages of low cost, light weight, renewability, biodegradability, easy processing, and high strength-to-weight ratio, plant fibers have been widely introduced into FDM 3D-printed composite samples in recent years [12,13,14]. Among these, wood flour (WF) has been used the most; for example, Kong et al. [15] prepared WF from poplar wood and then FDM 3D-printed WF/PLA biocomposite samples, using poly lactic acid graft maleic anhydride and silane coupling agent KH-550 as a compatibilizer and coupling agent, and poly butylenedioate-co-terephthalate and poly butylene succinate (PBS) as toughening agents. The modified composite had increased impact strength and storage modulus than in comparison to the unmodified one. In addition, the modified composite had an excellent molding effect, which was convenient for processing and industrial production. Nadir et al. [16] investigated the effect of printing layer thickness on the water absorption and mechanical properties of FDM 3D-printed WF/PLA composite materials. They found that an increased printing layer thickness led to increased water absorption and decreased thickness swelling, tensile, and flexural properties. The mechanical properties of recycled wood fibers/PLA/poly(hydroxyalkanoate) (PHA) biocomposites were found by Duigou et al. [17] to be strongly dependent on printing orientation (0 or 90°) during FDM 3D printing due to fiber anisotropy, and also on printing width (overlapping of filaments).

Resistance to aggressive environments, especially wet conditions, has been thought to be very important for materials [18]; hydrothermal aging of composite materials has thus been studied by many researchers. A series of results after hydrothermal aging of composites have been reported. Kanotha et al. [18] examined the moisture sorption, morphology, and some physical properties of WF/PLA composites after hydrothermal aging at various temperatures. It was revealed that increasing the temperature led to an increase in the rate of moisture uptake and percentage of crystallinity. Zeynal et al. [19] investigated the hydrothermal effect on the buckling behavior of glass/aramid/epoxy hybrid composites under distilled and seawater environments. The results indicated that the critical buckling load of the hybrid samples tested in a redried state increased less than the samples tested in a wet state; the composites aged in seawater had greater critical buckling loads than those aged in distilled water. In addition to these, the hydrothermal aging behaviors of some other composites, such as flax/PP [20], sisal/PLA [21], biocarbon/nylon [22], and flax/polyhydroxybutyrate(PHB) [23] composites have also been studied.

As a fully biodegradable aliphatic polyester, PBS has excellent mechanical properties and processability [24]. Blending PBS with PLA could increase the ductility of PLA without the loss of biodegradability [25,26].

Based on the aforementioned reasons, a WF/PBS/PLA biocomposite sample was developed by us as a novel feedstock for FDM 3D-printing technology, however, the sample aged under the influence of environmental factors during its storage and usage. Since then, the performance of all aspects reduced until the composite was unable to be used. Therefore, research on hydrothermal aging of FDM 3D-printed WF/PBS/PLA biocomposites and exploration of the function of the composite material after hydrothermal aging are of great significance to improving the application prospect of WF/PBS/PLA composite material. We FDM 3D-printed WF/PBS/PLA biocomposite samples in this study and investigated the effects of modification with a silane coupling agent and glycerol on the physico-mechanical properties of the printed samples, notably investigating the hydrothermal aging properties of these samples for the first time.

To sum up, a variety of natural-fiber-reinforced thermoplastic composites have been applied for FDM 3D-printing, and lots of composite materials have been hydrothermal aging tested, however, few studies have reported on the hydrothermal aging properties of FDM 3D-printed biocomposites. In this article, a novel filament was prepared using WF/PBS/PLA biocomposite and the hydrothermal aging properties of the samples was investigated for the first time, especially examining the effects of modification with a silane coupling agent and plasticizer on the properties of the printed samples before and after hydrothermal aging of the specimens was undertaken. The results from this research provided the experimental basis for practical applications of printed WF/PBS/PLA biocomposite materials.

## 2. Experimental Works

### 2.1. Materials and Reagents

PLA (American Nature Works Co., 3052D, Minnetonka, MN, USA) in pellet form was purchased from Suzhou Benfuzhong Plastic Import and Export Co., Ltd., China (Suzhou, China); PBS, HX-E201, was purchased from Anqing Hexing Chemical Corporation Limited, China (Anqing, China); WF, which was passed through a 60-mesh sieve, was kindly supplied by Nanjing Dayuan Ecological Construction Group (Nanjing, China); (3-aminopropyl)triethoxysilane (KH550), NH_2_(CH_2_)_3_Si(OC_2_H_5_)_3_, Grade AR, was purchased from Nanjing Chuanshi Chemical Co., Ltd., China (Nanjing, China); Glycerol, Grade AR, was purchased from Shanghai Aladdin Biochemical Technology Co., Ltd., China (Shanghai, China).

### 2.2. Pretreatment of WF with KH550

WF was treated with KH550, referring to the method reported by Kong et al. [15]. A 95% ethanol solution was prepared by mixing ethanol and distilled water, and its pH was adjusted to 3 by adding glacial acetic acid, then KH550 was added at a mass fraction of 4% to form the coupling agent solution for hydrolysis for 180 min. The dried WF and the coupling agent solution were weighed out at a ratio of 2:1, stirred evenly, dried for 30 min to volatilize most of the ethanol, vacuum dried for 30 min, and finally dried in a drying oven at 105 °C for 24 h to prepare KH550-modified WF, named as C-WF.

### 2.3. Sample Preparation

WF and C-WF were first dried at 105 °C to a constant mass. The PLA and PBS pellets were also dried at 60 °C to constant masses before processing to ensure the removal of any absorbed moisture. Before doing this work, when we previously FDM 3D-printed PBS/PLA blends and other natural fiber/PLA composites, it was found that the specimens containing 10 wt% PBS had the best comprehensive properties among all the printed blends, and the composites containing 10 wt% natural fiber could be printed smoothly and had good comprehensive properties. Thus, three kinds of composite materials were prepared with the following formulations.

- Untreated composite (coded as UN-C): weigh PLA, PBS, and WF by the mass fractions of 80%, 10%, and 10%, then mix all the components together.
- Glycerol-modified composite (coded as GM-C): weigh PLA, PBS, and WF by the mass fractions of 80%, 10%, and 10%, mix them together, then add glycerol as plasticizer into the mixture at 2% of the total mass of PLA, PBS, and WF, and mix once again.
- KH550-modified composite (coded as CM-C): weigh PLA, PBS, and C-WF by the mass fractions of 80%, 10%, and 10%, then mix all the components together.

The composite material was mixed in a high-speed mixer for 10 min, and the evenly mixed raw materials were put into a twin-screw extruder (SHJ-20, Nanjing Giant Machinery Co., Ltd., Nanjing, China) to melt and extrude pellets at 20 rpm and 130~160 °C. Then, the pellets were added into a single-screw extruder (KS-HXY, HUANXINYANG Electrical Equipment Co., Ltd., Suzhou, China) to prepare the mixed filament with a diameter of 1.75 ± 0.05 mm.

The prototypes of samples for different tests were built in computer-aided design software Cura_15.04.6 and then exported as an STL file. The STL file was further sliced into a data file by layers, which were made of Gcode. The STL file was sent to the FDM printer (MOSHU S108; Hangzhou SHINING 3D Technology Co., Ltd., Hangzhou, China) to prepare samples for performance testing. The printing parameters were set based on our previous work [27], i.e., the print temperature and speed were 220 °C and 50 mm/s, respectively, and the layer thickness was 0.1 mm.

### 2.4. Hydrothermal Aging Testing

Since each polymer has its own glass transition temperature and thermal stability, the hydrothermal aging conditions for the composites from various polymer matrices thus differed from one another [20,22,28,29]. Among these, the Chinese medicine residue (CMR)/PLA composites had similar components to WF/PBS/PLA. Therefore, the hydrothermal aging conditions reported in the literature [28] were referred for this study.

The hydrothermal aging test was carried out in distilled water under two conditions, i.e., 60 °C for 168 h, and 90 °C for 24 h. The changes that the specimens underwent in visual appearance, wettability, mass, and mechanical properties, as well as the thermal stability, were investigated.

### 2.5. Testing and Characterization

#### 2.5.1. FTIR Analysis

Fourier-transform infrared (FTIR) spectroscopy of wood flour was carried out using a Bruker attenuated total reflection FTIR spectrometer (VERTEX 70, Bruker Optics, Ettlingen, Germany). The IR spectrum was recorded in a range from 400 cm^−1^ to 4000 cm^−1^, with a resolution of 4 cm^−1^ at 32 scans/min. Before this, the wood flour was mixed with potassium bromide (KBr) by a mass ratio of 1:100 and then compressed into tablets.

#### 2.5.2. Wettability Testing

Wettability testing was carried out referring to the ASTM D 724 standard [30] testing method and the methods reported in the literature [31,32]. A contact angle instrument (DSA100; KRÜSS GmbH, Borsteler Chaussee, Germany) was employed to test the contact angles of a distilled water drop on the surface of each FDM 3D-printed biocomposite sample at room temperature. A 5 µL droplet of distilled water was dropped onto the surface and kept there for 15 s, and then the contact angles from the images were measured at different points. Ten specimens were used for each sample.

#### 2.5.3. Water Uptake Analysis

The water absorption property of the samples was investigated through the moisture absorption testing. The percentage of moisture uptake (wu%) was estimated with the following equation:

(1) $$wu(\%)=\frac{m_{t}-m_{0}}{m_{0}}\times 100\%$$

where m_0_ was the mass of the samples after immersion in water at room temperature for 15 d, at 60 °C for 168 h, or at 90 °C for 24 h. m_t_ was the initial dry mass of the specimens. An average value of at least five measurements was calculated to obtain the reported results.

#### 2.5.4. Weight Loss

The samples tested at 60 °C or 90 °C in Section 2.5.3 were taken out of the water, cleaned, and dried in an electric thermostatic drying oven to a constant mass, then weighed. The weight loss (WL) of each sample was calculated by weight variation between the samples before immersion and after drying using the following formula:

(2) $$wl(\%)=\frac{w_{0}-w_{t}}{w_{0}}\times 100\%$$

where, wl was the weight loss rate, w_0_ was the mass recorded before immersion in water, and w_t_ was the drying mass recorded after immersion.

#### 2.5.5. Mechanical Testing

The tests for tensile properties were conducted in air at room temperature after hydrothermal aging to evaluate the degradation of mechanical properties. Loading was introduced by means of a 20 kN capacity testing machine (E44.304, MTS Industrial Systems (China) Co., Ltd., Shenzhen, China) and the constant cross-head rate for the test was 10 mm/min, referring to the ASTM D 638 standard [33] testing method.

#### 2.5.6. Morphological Characterization (SEM)

The morphology of the cross-sectional surface of the printed samples after tensile testing was observed by a Hitachi SU 8010 field-emission scanning electron microscope (Hitachi Corporation, Tokyo, Japan) at an accelerating voltage of 3 kV. All of the fracture surfaces were first sprayed with a thin layer of gold to avoid any electrostatic charge and poor resolution during the scanning examination.

#### 2.5.7. Thermal Stability

TGA analysis was conducted using a thermogravimetric analyzer (NETZSCH-Gerätebau GmbH, Selb, Germany) to assess the thermal stability of the printed samples, referring to the methods reported in the previous literature [31,34]. Each sample (approximately 5~10 mg) was placed in a standard crucible and heated from 20 °C to 550 °C at a 20 K/min heating rate and a flow rate of 20 mL/min. The sample’s mass loss (TG) curve and derivative mass loss (DTG) curve were drawn. The thermal stability of the sample was evaluated by the initial decomposition temperature (T_i_) of the samples in the TG curve and the maximum thermal weight loss rate temperature (T_max_) in the DTG curve.

## 3. Results and Discussion

### 3.1. FTIR Analysis

**Figure 1:**
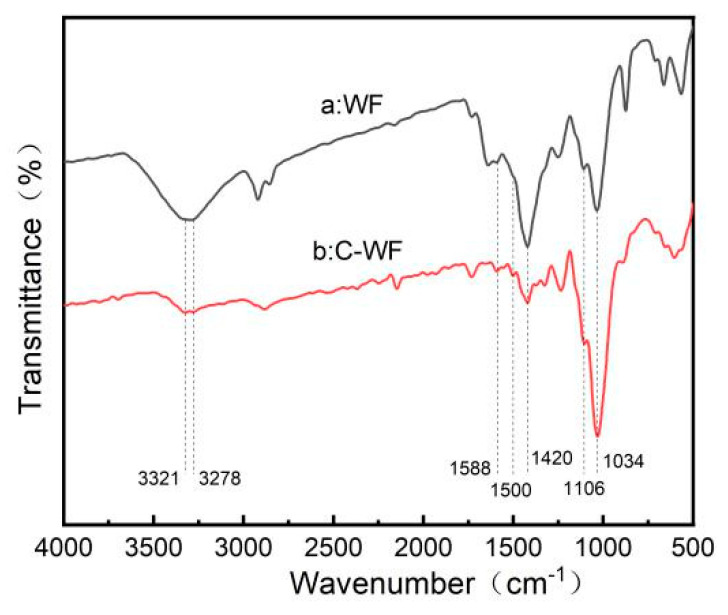
Fourier-transform infrared (FTIR) spectra of WF and C-WF.

Figure 1 shows the FTIR spectra of the surfaces of WF and C-WF.

Some changes occurred to the spectra after WF was treated with KH550. The wide band around 3300 cm^−1^ in WF is due to the stretching vibration of hydroxyl groups; for C-WF, however, the wide band is replaced by two peaks at around 3321 cm^−1^ and 3278 cm^−1^, corresponding to the absorbance of hydroxyl groups from WF and amino groups from KH550 [35]. The absorbance by -OH groups was found to be weakened dramatically; this might be due to the reduction in hydroxyl groups resulting from the reaction between the -OH groups and KH550. The absorbance by the amino groups was not obvious either; this might be because of the poor concentration of KH550 on the fiber surfaces. Meanwhile, this was affected by the stretching vibration modes derived from the unreactive hydroxyl on WF surfaces in this region [36]. The appearance of a new absorbing peak emerged at around 1500 cm^−1^, and the slight increment of intensity for the band at 1588 cm^−1^ in C-WF might be the result of the N-H bending and absorbing vibration [36]. The absorbance around 1420 cm^−1^ originated from aromatic skeletal vibrations from lignin in WF and was associated with CH and CH_2_ vibrations [37]. After KH550 treatment the absorbance became weakened, which might be caused by the interference from the reaction between the silane and the branches on the aromatic skeletal vibrations. The slight increment of intensity for the band at approximately 1106 cm^−1^ in C-WF compared to that in WF might be attributed to the formation of a covalent -Si-O-C- bond as a result of the grafting reaction between silanols and wood fiber under heating conditions [38]. For WF, a peak around 1034 cm^−1^ was observed due to the stretching of C-O and C-O-C vibrations of cellulose. After being treated with KH550, this peak was enhanced due to the stretching of Si-O-C and Si-O-Si bonds around 1030–1200 cm^−1^; the result also reveals silane presence on the C-WF surfaces [39]. All of the results as depicted above prove that WF had been successfully modified by KH550 through a chemical bonding process instead of only physical absorption.

### 3.2. Visual Appearance

**Figure 2:**
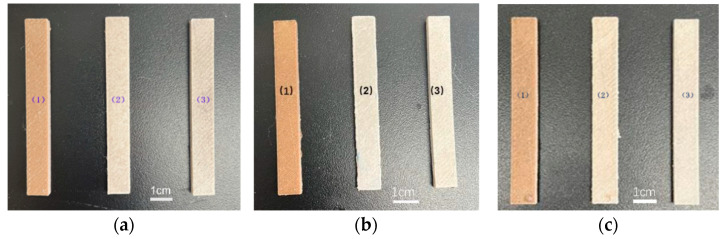
Visual appearances of the samples under different hydrothermal aging conditions: (**a**) UN-C; (**b**) GM-C; (**c**) CM-C.

A significant change in color of the natural reinforced composites was usually observed during hydrothermal aging [20]. Figure 2 displays the resulting visual appearances of the printed samples. The samples labeled (1), (2), and (3) in each picture represent those before aging and after aging at 60 °C and at 90 °C, respectively. All of the samples before aging were light yellow in appearance, while all the surfaces became whitened after thermal aging. Kanotha et al. [18] reported the same effects for composites of PLA and WF submerged at 47 °C. When the hydrothermal temperature increased from 60 °C to 90 °C, the surfaces of all of the samples became whitened to almost the same extent, indicating that the increase in aging temperature had the same effect on the visual appearances of the samples as the prolonging of the aging time.

### 3.3. Wettability

**Figure 3:**
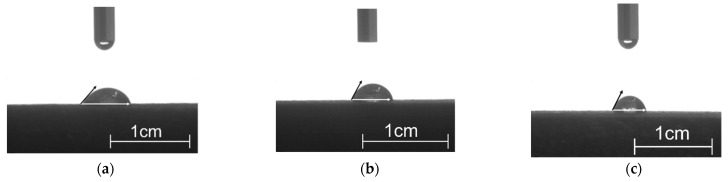

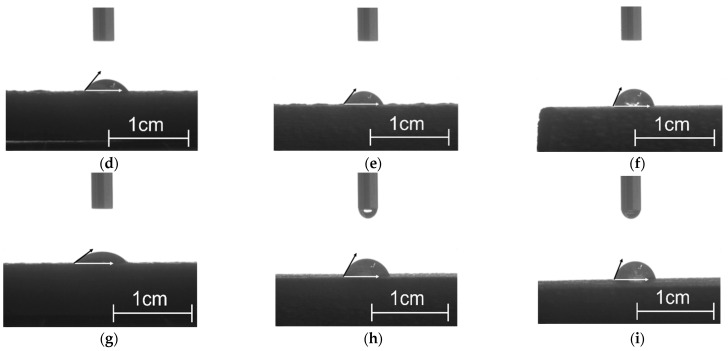
The shapes of the water droplets on the surface of the printed samples: (**a**) UN-C before aging; (**b**) UN-C after hydrothermal aging at 60 °C for 168 h; (**c**) UN-C after hydrothermal aging at 90 °C for 24 h; (**d**) GM-C before aging; (**e**) GM-C after hydrothermal aging at 60 °C for 168 h; (**f**) GM-C after hydrothermal aging at 90 °C for 24 h; (**g**) CM-C before aging; (**h**) CM-C after hydrothermal aging at 60 °C for 168 h; (**i**) CM-C after hydrothermal aging at 90 °C for 24 h.

Figure 3 shows the shapes of the water droplets on the surfaces of the printed samples, and the corresponding contact angles are tabulated in Table 1. It could be observed that the surface wettability of the printed samples differed with different modification methods and aging conditions, although all the contact angles were smaller than 90°, indicating that they were all hydrophilic. The contact angle of UN-C before aging was 63.5°, while this decreased to 58.8° by 7.40% for GM-C, and to 55.0° by 13.39% for CM-C, meaning that the hydrophilicity increased after modification. Where the effect of the hydrothermal aging on wettability was concerned, all of the contact angles clearly increased, indicating that all of the composites became more hydrophobic after aging. When WF as a solid phase and the PBS/PLA blend as a liquid phase were applied to FDM 3D-printed biocomposite materials, the surface free energy of the fluid should have been equal to or lower than that of the solid. A greater difference in the surface free energies between the two phases would make it easier for WF to wet the surface of the PBS/PLA blend and form a good interface fusion [15]. The decrease in the contact angles of the water droplets on the surfaces of the biocomposite materials because of modifications, and the increase because of hydrohermal aging, thus reflected the interfacial compatibilities to some extent, i.e., modifications improved the interfacial compatibility, the opposite was true for hydrothermal aging.

After hydrothermal aging at 60 °C for 168 h, the contact angles of UN-C, GM-C, and CM-C increased by 13.39%, 13.44%, and 20.0%, respectively. In this situation, the contact angles of GM-C and CM-C were almost the same, and were still much smaller than that of UN-C, showing that the modification improved the anti-hydrothermal aging properties at lower temperatures. A similar phenomena happened to those samples after hydrothermal aging at 90 °C for 24 h. When comparing the wettability of the samples that were hydrothermally aged at 60 °C and 90 °C, the contact angles of all the samples clearly increased under higher temperature conditions, though the immersion time was shorter than that at 60 °C. Thus, it could be concluded that the temperature of hydrothermal aging had a greater effect on the wettability of the samples than the immersion time. The change in wettability implied the alternation of the internal structure of the sample, meaning that a higher hydrothermal temperature would destroy the construction of the samples more significantly.

### 3.4. Water Uptake and Mass Retention

**Figure 4:**
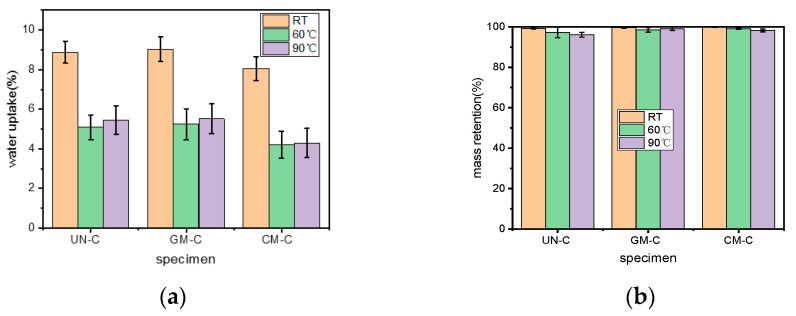
Mass retention of the samples: (**a**) water uptake; (**b**) mass retention.

The experimental values of the water uptake and mass retention of the composite samples with respect to the hydrothermal aging are presented in Figure 4. As shown in Figure 4a, the water uptake of UN-C, GM-C, and CM-C immersed in water at room temperature for 15 d was 8.88%, 9.02%, and 8.06%, respectively. The modification of WF by KH550 enhanced the bonding between the fiber and the resin [15], leading to the reduction in water uptake of the samples. The greater moisture absorption of GM-C than UN-C might be due to the hydrophilicity of the hydroxyl groups in the structure of glycerol itself. After hydrothermal aging, the water uptake of all the samples clearly decreased; this was consistent with the results of the wettability testing. After hydrothermal aging, all of the samples turned out to be more hydrophobic, as evidenced in Figure 3 and Table 1. As a consequence, the water uptake decreased. In addition, the water uptake of each sample after being hydrothermally aged at 60 °C for 168 h was not very different from that of the sample after being aged at 90 °C for 24 h, meaning that the thermal equivalence of time could be applied to the investigation of water uptake during hydrothermal aging of polymer materials.

Regarding the mass retention, no great changes happened to the samples that had been immersed in water at room temperature, as illustrated in Figure 4b; the mass was maintained by more than 99% for each sample, meaning that a lower temperature had little effect on the mass change. After being hydrothermally aged at 60 °C for 168 h or 90 °C for 24 h, however, mass was clearly lost for all of the samples, especially for UN-C. The UN-C mass retentions were only 95.22% and 94.14%, respectively, lower than those of GM-C by 1.34% and 2.90%, and those of CM-C by 1.89% and 2.11%. The results of mass retention also confirmed that hydrothermal aging would destroy the internal structures of the samples, and modification could enhance the resistance of the samples to the hydrothermal aging.

### 3.5. Tensile Performances Analysis

**Figure 5:**
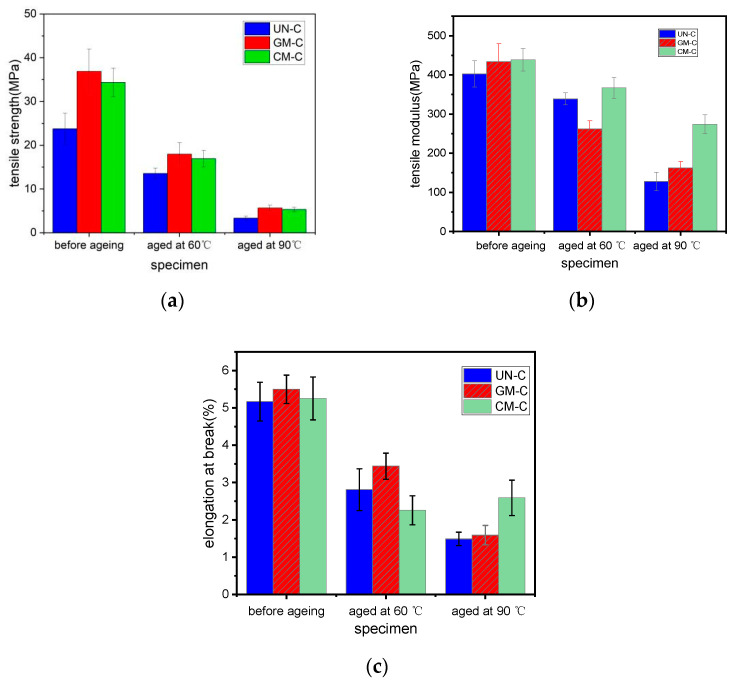
Tensile properties of the printed samples: (**a**) tensile strengths; (**b**) tensile moduli; (**c**) elongation at break.

The effects of modification and hydrothermal aging conditions on the tensile properties of the printed samples were investigated. The results are shown in Figure 5. It could be inferred that modification with glycerol and KH550 could dramatically improve the tensile strength of UN-C from 23.76 MPa to 36.86 MPa and 34.35 MPa, by 55.13% and 44.57%, respectively. Meanwhile, the tensile modulus could be improved from 402.74 MPa to 434.15 MPa and 438.61 MPa, by 7.80% and 8.90%, respectively, and the elongation at break by 9.48% and 6.38%, respectively. The improved tensile properties of GM-C compared to UN-C may be attributed to the plasticizing action by glycerol, and those of CM-C to the enhancement of the interfacial bonding between C-WF and the matrix [40]. After being hydrothermally aged at 60 °C for 168 h, the tensile strengths of the samples reduced to 13.55 MPa, 17.96 MPa, and 16.92 MPa; the tensile modulus decreased to 338.88 MPa, 262.38 MPa, and 366.93 MPa; and the elongation at break dropped to 2.81%, 3.44%, and 2.43%, respectively. After being aged at 90 °C for 24 h, a great decrease in tensile strength by 85.98%, 84.64%, and 84.57% happened to UN-C, GM-C, and CM-C, respectively. The tensile strengths of UN-C, GM-C, and CM-C were now only 3.33 MPa, 5.66 MPa, and 5.30 MPa; meanwhile, the tensile modulus reduced to 128.1 MPa, 162.39 MPa, and 274.18 MPa; and the elongation at break dropped significantly to 1.49%, 1.59%, and 2.79%, respectively. The tensile properties were all much poorer than those of the samples aged at 60 °C, implying that the internal structures of the samples were destroyed more heavily after hydrothermal aging at higher temperature. Bao [28] once investigated the hydrothermal aging properties of CMR/PLA biocomposites; the tensile strengths of the specimens after hydrothermal aging at 90 °C for 15 h and 60 °C for 8 days were 2.48 MPa and 4.32 MPa. It was seen from this that the WF/PBS/PLA specimens in our study showed slightly improved hydrothermal-aging-resistant properties than CMR/PLA samples when only the tensile strengths were taken into consideration. Moreover, a similar considerable loss of stiffness and strength after hydrothermal aging had also been observed in other biocomposites such as flax fabric/PHB biocomposites [23]. The reduction in mechanical properties of natural-fiber-reinforced composites after hydrothermal aging was thought to be synergically caused by moisture and temperature [41]. In this situation, however, the tensile strengths of GM-C and CM-C were still greater than that of UN-C by 75.23% and 59.16%, and the modulus by 26.77% and 114.04%, respectively, meaning that modification with both glycerol and KH550 could improve the resistance of the samples to the hydrothermal aging.

From Figure 5, it was found that the elongation at break for each specimen at any aging stage was lower than 6%. Especially after hydrothermal aging, the elongation at break of all of the samples became much smaller, indicating that UN-C, GM-C, and CM-C all broke in a brittle manner.

Comparing the tensile properties of GM-C and CM-C after hydrothermal aging, the results from Figure 5a,b show that there was little difference in their tensile strengths, however, the tensile modulus of CM-C was much greater than that of GM-C, either after being aged at 60 °C or at 90 °C, suggesting that modification with KH550 could improve the anti-hydrothermal aging properties of the samples better than that with glycerol.

### 3.6. Cross-Sectional Morphologies

**Figure 6:**
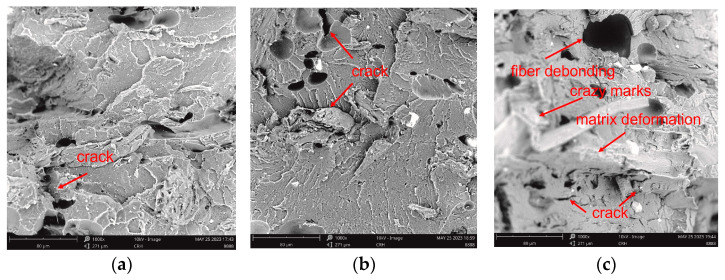

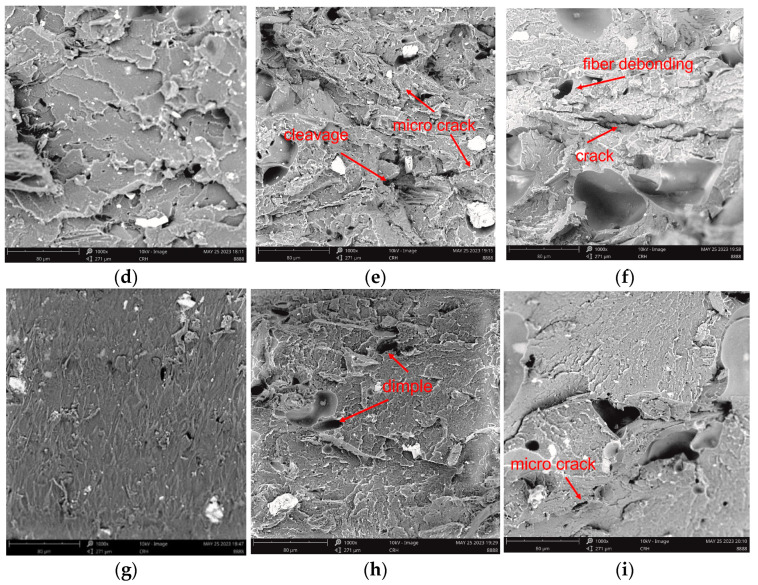
Cross-sectional morphologies of the printed samples: (**a**) UN-C before aging; (**b**) UN-C after hydrothermal aging at 60 °C for 168 h; (**c**) UN-C after hydrothermal aging at 90 °C for 24 h; (**d**) GM-C before aging; (**e**) GM-C after hydrothermal aging at 60 °C for 168 h; (**f**) GM-C after hydrothermal aging at 90 °C for 24 h; (**g**) CM-C before aging; (**h**) CM-C after hydrothermal aging at 60 °C for 168 h; (**i**) CM-C after hydrothermal aging at 90 °C for 24 h.

SEM images at ×1000 magnification after fracture obtained of 3D-printed materials at different hydrothermal aging temperatures are shown in Figure 6. It could be observed that the fracture surfaces of all of the samples were generally flat and no natural fibers were pulled out of the matrix. All the specimens failed due to intra-layer debonding resulting in a brittle failure mode. Such a smooth surface and brittle behavior were also reported by the literature [42], and hydrothermal aging did not change the breaking mechanisms.

For UN-C, Figure 6a illustrates that there was a poor interfacial bonding between fiber and matrix, and some cracks could even be observed in this situation. This was mainly because the hydrophilic WF and hydrophobic polymer matrix belonged to two incompatible systems [40]. This brittle fracture behavior of UN-C was inconsistent with that of printed hemp/PLA biocomposite observed by Sofiane et al. [43] using SEM. For the hemp/PLA biocomposite, interfacial decohesion happened between the PLA matrix and the hemp fibers as a main deformation mechanism, contributing to the failure of the printed material. In addition, the crack path inside UN-C was not fixed, which may have been caused by random fiber distribution in the matrix [44]. After hydrothermal aging at 60 °C, more cracks happened. When the aging test was carried out at 90 °C, some fibers debonded from the matrix, and the matrix deformed severely and cracked more seriously. Meanwhile, crazing marks could be observed. All of the phenomena indicated that UN-C was easy to age hydrothermally.

Figure 6d–f demonstrates the cross-sectional morphologies of GM-C. The interfaces became more homogeneous and a little rough; the fiber and the matrix bonded well before aging. The reason may be that WF could adhere to the polymer matrix more tightly after glycerol was applied as a toughener. When a load was exerted on the specimen, WF would deform simultaneously with the matrix, and then they would be broken together. This meant that the addition of glycerol had not obviously changed the brittle fracture characteristics of WF/PBS/PLA biocomposites [45], which was consistent with the results of the tensile testing. After being hydrothermally aged at 60 °C, some cleavages and even micro cracks appeared, but not as obviously as in UN-C. After being hydrothermally aged at 90 °C, some cracks also appeared and some fibers also debonded from the matrix, however, the interfacial bonding was much better than that of UN-C after aging, showing that modification with glycerol could not only enhance bonding among all the components, but it could also improve the anti-hydrothermal aging properties of the biocomposites. 

The fracture surfaces of the samples modified by KH550, i.e., CM-C, are illustrated in Figure 6g–i. The SEM images revealed that the interface became more uniform than that of GM-C, and the existence of WF in the structure of tensile fractures was not observed. The observation of the fractures allowed for the conclusion that WF particles were homogeneously incorporated into the polymer matrix [46], indicating that the modification with KH550 could improve the bonding between WF and the matrix better than modification with glycerol. After hydrothermal aging at 60 °C, CM-C had much better interfacial bonding than UN-C and even than GM-C; only some dimples were produced. This improvement in hydrothermal aging properties using a coupling agent was also examined in research by Gil-Castell et al. [21]; in their work, the presence of a coupling agent would reduce the delamination between the fibers and polymeric phase. After being aged at 90 °C, few fibers debonded from the matrix and micro cracks happened partially. It was evidenced by the SEM pictures that both the modifications with glycerol and KH550 improved the bonding between the fibers and the matrix. Meanwhile, the composites became more resistant to hydrothermal aging, and modification with KH550 had more pronounced effects on the anti-hydrothermal aging properties.

### 3.7. Thermogravimetric Analysis

**Figure 7:**
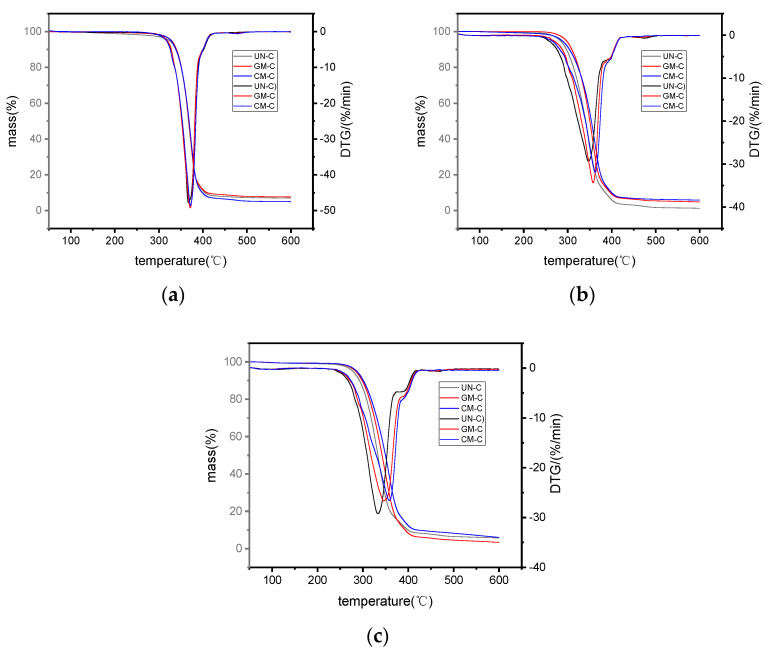
TGA and DTG curves of the printed samples: (**a**) before aging; (**b**) after hydrothermal aging at 60 °C for 168 h; (**c**) after hydrothermal aging at 90 °C for 24 h.

Understanding the thermal stability of a biocomposite is helpful for ascertaining its state of chemical degradation during hydrothermal aging [21]. Thermal stability is usually assessed by means of thermogravimetric analysis (TGA) [7,21,27]. Figure 7 presents the TGA and its first-derivative thermogravimetric (DTG) during the decomposition of the printed WF/PBS/PLA samples before aging and after hydrothermal aging at 60 and 90 °C. The corresponding characteristic thermal degradation parameters of the thermogravimetric curves are given in Table 2. It indicated in Figure 7a and Table 2 that all of the composites exhibited similar thermal degradation curves under identical conditions; the T_i_ and T_p_ values of the three samples were all around 347 °C and 371 °C. After being hydrothermally aged at 60 °C for 168 h, GM-C had the greatest T_i_ and T_p_ values of 321.0 °C and 362.4 °C, 2.2 °C and 4.5 °C more than those of CM-C, and 15.8 °C and 15.1 °C more than those of UN-C, respectively. For the samples being hydrothermally aged at 90 °C for 24 h, the T_i_ and T_p_ values of GM-C decreased to 309.7 °C and 358.1 °C, respectively, which were still the greatest among all the samples; those of CM-C reduced to 304.3 °C and 347.3 °C, and those of UN-C to 299.0 °C and 333.5 °C. The results showed that modification with glycerol or KH550 could improve the thermal stability of the samples during the hydrothermal aging test, and modification with glycerol had greater effect on the improvement than with KH550. In addition, a higher temperature would worsen the thermal stability of the samples more heavily, though the test time was shorter, indicating that the hydrothermal aging temperature had a greater effect on the thermal stability of the samples when compared to the testing time, which was consistent with the results of the mechanical testing.

## 4. Conclusions

This study first prepared WF/PBS/PLA biocomposite samples by FDM 3D-printing technology, then carried out the hydrothermal aging test on the samples, and the changes in the properties of the samples due to aging were studied. The following conclusions can be drawn.

Modification with glycerol or KH550 could improve the interfacial bonding among different components in the samples, and increased the hydrophilicity as well as the tensile strengths of the printed specimens before and after hydrothermal aging. Modification could improve the thermal stability of the samples during the hydrothermal aging test, and the hydrothermal aging temperature had a greater effect on the thermal stability of the samples when compared to the testing time.

After hydrothermal aging, the surfaces of all of the samples, no matter whether they were modified or not, became whitened from their virgin light yellow color.

After hydrothermal aging, all of the composites became more hydrophobic; aging at 90 °C made all of the samples have greater contact angles than aging at 60 °C.

Hydrothermal aging would cut down the moisture absorption and mass retention of all the samples. Modification with glycerol or KH550 could make the samples achieve greater mass retention after hydrothermal aging than unmodified samples.

As a conclusion, hydrothermal aging will destroy the internal structure of FDM 3D-printed WF/PBS/PLA samples and worsen their properties, and a higher hydrothermal temperature will destroy the construction of the samples more significantly. Modification with glycerol or KH550 can improve the resistance of the samples to hydrothermal aging.

## Figures and Tables

**Table 1:** Contact angle of distilled water on surface of the printed samples.

Sample Codes	Before Aging (°)	Aged at 60 °C for 168 h (°)	Aged at 90 °C for 24 h (°)
UN-C	63.5	72.0	80.9
GM-C	58.8	66.7	77.9
CM-C	55.0	66.0	75.4

**Table 2:** Thermogravimetric analysis information of different specimens.

Sample Code	Unaged		Aged at 60 °C		Aged at 90 °C	
	Ti/°C	T_p_/°C	Ti/°C	T_p_/°C	Ti/°C	T_p_/°C
UN-C	347.4	371.8	305.2	347.3	299.0	333.5
GM-C	346.7	370.3	321.0	362.4	309.7	358.1
CM-C	347.0	371.3	318.8	357.9	304.3	347.3

## Data Availability

Not applicable.

## Sample Availability

Samples of the compounds are not available from the authors.
